# The Rare Bone Disease TeleECHO Program: Leveraging Telehealth to Improve Rare Bone Disease Care

**DOI:** 10.1007/s11914-020-00595-2

**Published:** 2020-06-08

**Authors:** Laura L. Tosi, Elmer N. Rajah, Michael H. Stewart, Austin P. Gillies, Tracy S. Hart, E. Michael Lewiecki

**Affiliations:** 1 Division of Orthopaedics & Sports Medicine, Children’s National Hospital, 111 Michigan Ave NW, Washington, DC 20010 USA; 2 The George Washington University School of Medicine and Health Sciences, 2310 I St. NW, Washington, DC 20052 USA; 3 Osteogenesis Imperfecta Foundation, 804 W Diamond Ave #210, Gaithersburg, MD 20878 USA; 4 New Mexico Clinical Research & Osteoporosis Center, 300 Oak St NE, Albuquerque, NM 87106 USA

**Keywords:** Force multiplier, Telementoring, Community of practice

## Abstract

**Purpose of Review:**

Rare bone diseases constitute ~ 5% of all known rare diseases and can require complex, multidisciplinary care. Advancing access to current medical knowledge is an important strategy for improving care for rare bone diseases throughout the world. To support this goal, the Rare Bone Disease Alliance launched the Rare Bone Disease TeleECHO in 2019.

**Recent Findings:**

The Rare Bone Disease TeleECHO is a monthly video teleconference that fosters a collegial community of practice and opportunities for active learning through interactive case-based learning. TeleECHO relies on a hub-and-spoke model, where medical professionals at the “hub” provide support and expertise for other healthcare providers, or the “spokes”. Evidence of the global reach of the program as well as qualitative feedback from registrants supports the need for rare bone disease education and the value of the TeleECHO model.

**Summary:**

The Rare Bone Disease TeleECHO helps meet the challenge of disseminating rapidly expanding rare bone disease knowledge by leveraging telehealth.

## Introduction

Rare bone diseases constitute an important fraction (~ 5%) of all known rare diseases and typically require complex, life-long management [1]. Even specialists whose practices focus on bone health and skeletal dysplasias may never see many of the 461 currently described skeletal disorders during their careers, making it difficult to diagnose and manage some of these patients correctly. Moreover, rapid advances in genomic sequencing and gene therapy are fundamentally changing the diagnosis and management of all rare disease.

The Rare Bone Disease Alliance (RBDA), a program of the Osteogenesis Imperfecta Foundation (OIF), is a coalition of twelve rare bone disease advocacy organizations, clinicians, and researchers focused on educating medical professionals, expanding research, and assisting patients and communities affected by rare skeletal diseases. It believes that expanding access to continuing medical education on rare bone disease topics for bone health clinicians and researchers is a critical goal for improving the care of people with rare diseases worldwide. The RBDA collaborated with Project Extension for Community Healthcare Outcomes (Project ECHO), to facilitate access to post-graduate rare bone disease medical education. Project ECHO originated as a way to disseminate medical knowledge to isolated primary care physicians and improve clinical outcomes. The Rare Bone Disease TeleECHO adapts this model by partnering a core faculty of rare bone disease specialists and topic experts with rare bone disease clinicians worldwide so they can share advances in research and best practices. This report describes the opportunities the Rare Bone Disease TeleECHO offers for improved care of patients with rare bone disease, regardless of where they reside. It also outlines how the ECHO model functions, because it is applicable to many other disease groups.

## The Echo Model

Project ECHO was conceived by Sanjeev Arora, M.D., at the University of New Mexico in 2003 in response to the highest per capita rate of viral hepatitis in the USA. Hepatitis C was only being treated in 1600 out of an estimated 34,000 patients in the state. Arora was one of a few doctors who specialized in treating hepatitis C. He realized that the root of the problem was not a shortage of doctors or resources, but rather a monopoly of medical knowledge inside the university setting [2••].

Arora’s solution was a platform for collaborative medical practice—Project ECHO. Project ECHO links primary care clinicians to specialist care teams at large hospitals through an interactive teleconference. Specialists share their medical knowledge and expertise through brief didactic presentations, and then clinicians from multiple sites present de-identified patient cases to the specialist teams and discuss options for enhancing diagnosis and treatment. Case presentations are not delivered in a “Q&A” format for experts, but rather as a conversation among providers. The aim of this discussion is for experts to guide and support other providers and foster opportunities for active learning.

**Fig. 1:**
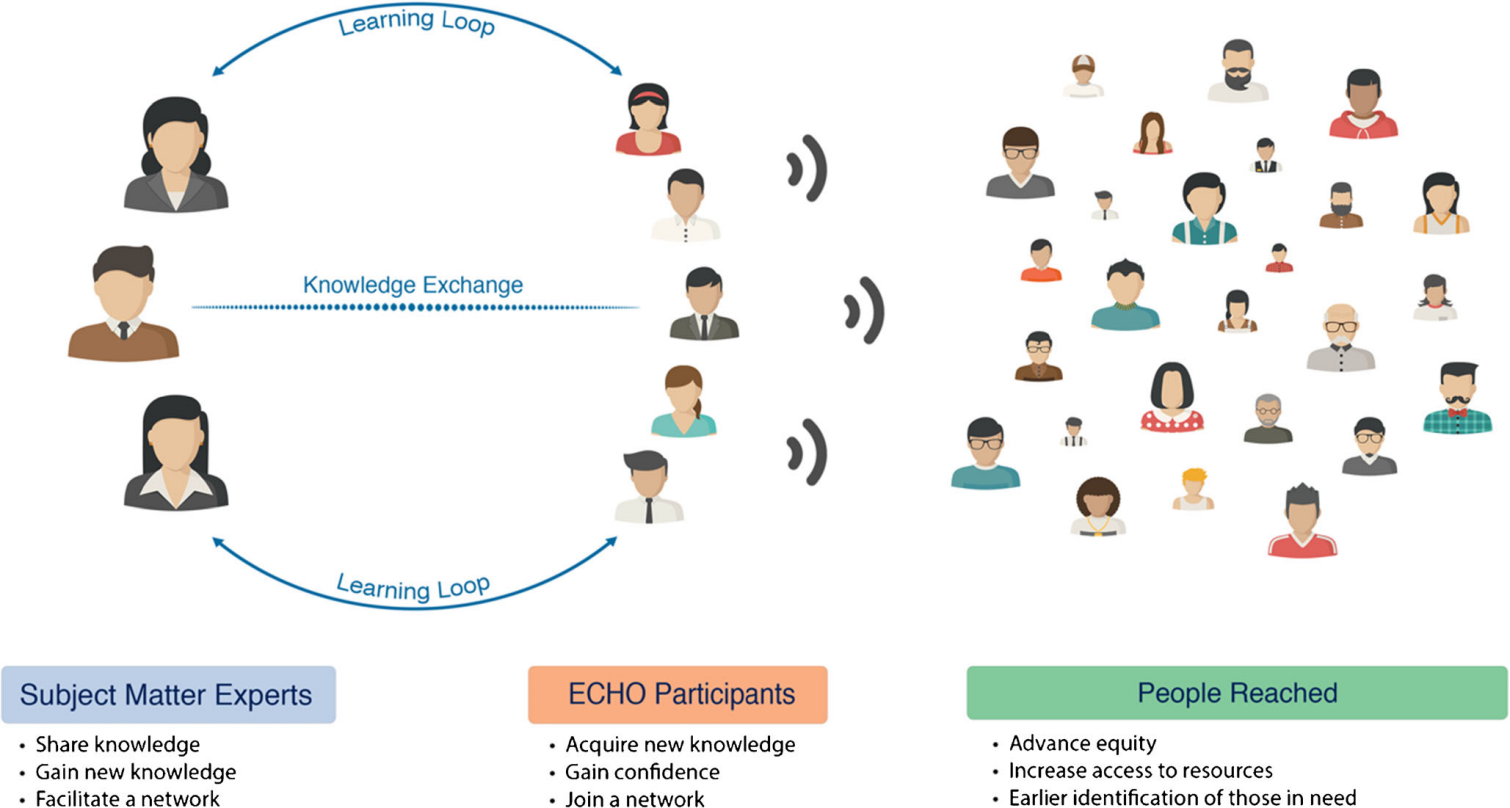
Force Multiplier. The ECHO model serves as a force multiplier. A few specialists at the “hub” have a much larger on patient care and outcomes than traditionally permitted through telemedicine (contains artwork made by Eucalyp on www.flaticon.com)

Project ECHO relies on a “hub-and-spoke” model, where specialists at the centre (the “hub”) can engage with and assist a broader audience at the periphery (the “spokes”). A team of specialists trains and supports multiple community-based providers, who in turn treat a much larger population of patients than traditional telemedicine permits. The ECHO model is not telemedicine in which one clinician manages one patient from a remote location, but rather a “force multiplier” that amplifies access to and the impact of highly trained specialists (Fig. 1). The ECHO model is designed to break down hierarchies and promote active participation and discussion.

## Adapting the TeleECHO Model to Support Rare Bone Disease

Bone Health TeleECHO programs have been established for osteoporosis, Ehlers-Danlos Syndrome, and Fracture Liaison Services over the past few years, but no similar program existed for rare bone diseases [3••]. The leaders of the RBDA recognized that Project ECHO could be adapted as a tool to educate specialist clinicians and enhance care to patients living with rare bone disease, and launched the Rare Bone Disease TeleECHO in August of 2019.

The Rare Bone Disease TeleECHO organizational structure consists of a faculty chair, medical faculty, and a program manager. The medical faculty “hub” consists of six rare bone disease specialists who develop the annual agenda, identify speakers, and support topic experts during the case discussions portion of the ECHO sessions. Speakers for the didactic portion of the meeting are chosen for their content expertise. The program manager is responsible for administrative and operational tasks.

Although every ECHO clinic is licenced under Project ECHO and remains true to the traditional ECHO principles, four innovations characterize the Rare Bone Disease TeleECHO.

- First, while most ECHO programs focus on a single disorder, the Rare Bone Disease TeleECHO alternates between programs focused on specific rare bone disorders and programs focused on differential diagnosis. Table 1 displays the topics to be covered during Year One. The plan is to present every disease entity included in the Rare Bone Disease Alliance for discussion during the first 2 years of the ECHO program. Including differential diagnosis topics also allows us to cover an even broader range of disorders.
- Second, the Rare Bone Disease TeleECHO uses an expanded faculty model. Project ECHO recommends a 3–4 member hub faculty. Rare Bone Disease TeleECHO sessions are supported by the invited speaker, the TeleECHO faculty, and interested national and international experts, who fosters cutting edge analysis of the cases presented.
- Third, the Rare Bone Disease TeleECHO is trying to reach medical faculty beyond the major research centres who do not have the benefit of a strong connection to the rare bone disease research enterprise. It is not primarily seeking community practitioners, but rather ensuring that the broad range of rare bone disease specialists (geneticists, endocrinologists, orthopaedic surgeons, as well as their support staff) is able to optimize diagnosis and care through the support of telementoring. In addition, our speakers have been very gracious about responding to participant queries following ECHO presentations.
- Fourth, the Rare Bone Disease TeleECHO aims to provide recent research results in a field where advances are accumulating rapidly. Its focus is prompt research transmission versus standard protocols.

**Table 1:** Rare Bone Disease TeleECHO Agenda Year One

8/1/2019	Genetic Testing in the Diagnosis of Rare Bone Disease	Eric T. Rush, MD – University of Kansas Medical Center
9/5/2019	OI Dominant vs Recessive: Impact on Treatment	Reid Sutton, MD – Baylor College of Medicine
10/3/2019	Hypocalcemia	Dolores Shoback, MD – University of California, San Francisco
11/7/2019	Fibrous Dysplasia/McCune Albright Syndrome	Michael Collins, MD – National Institutes of Health, NIDCR
12/5/2019	Non-Accidental Trauma	Peter Byers, MD – University of Washington Medicine
1/2/2020	XLH Disorders	Thomas Carpenter, MD – Yale Medicine
2/6/2020	Diagnostic Approach to the Child with a Skeletal Dysplasia	Julie Hoover-Fong, MD, PhD – Johns Hopkins Medicine
3/5/2020	Hypophosphatasemia	Michael Whyte, MD – Shriners Hospital for Children
4/2/2020	Evaluation of the Child with Rickets	Erik Imel, MD – Indiana University School of Medicine
5/7/2020	Osteopetrosis	Michael Econs, MD – Indiana University School of Medicine
6/4/2020	Fibrodysplasia Ossificans Progressiva	Edward Hsiao, MD, PhD – University of California, San Francisco
7/2/2020	Management of Pregnancy & Delivery in the Patient with a Skeletal Disorder	Deborah Krakow, MD – University of California, Los Angeles

## Operating the Program

Prior to starting an ECHO program, prospective leaders are required to attend a 3-day immersion training in Albuquerque, NM, USA. Project ECHO faculty train attendees on the core tenets of the ECHO model: moderating sessions, methods for evaluation, and using the Zoom platform. Attendees are also afforded the opportunity to observe and participate in mock ECHO sessions. Following immersion training for the faculty chair and the TeleECHO manager, our medical faculty convened to set goals and objectives, create a curriculum for Year One, and choose featured speakers.

In the weeks before a scheduled ECHO session, the program manager advertises the meeting through various channels, including registration lists, the American Society for Bone and Mineral Research (ASBMR) and OIF websites, and professional networks. Along with the meeting announcement, the manager encourages attendees to submit cases for presentation. In the weeks preceding the call, both the faculty chair and the program manager hold a rehearsal session with the featured speaker to practice operating basic functionalities and ensure that the speaker understands how a TeleECHO event differs from a traditional webinar. The program manager and faculty chair also review the case presentations to ensure content quality and that all patient identifiers are removed, as well as provide tips and support for new presenters.

Rare Bone Disease TeleECHO sessions are hosted via Zoom video teleconferencing, which is offered at no cost to organizers and attendees through Project ECHO. Zoom has a simple user interface and can be accessed from a phone or computer with internet access. Meeting data is end-to-end encrypted and the platform complies with Health Information Protection and Portability Act (HIPAA) standards regarding access, audit controls, and protection of health information. Zoom also has built-in recording functionality that allows for didactic presentations to be uploaded to the OIF website for individuals with scheduling conflicts to watch at a later date (case presentations are not recorded to protect patient privacy). The simple user interface, ease of use, and a wide array of host tools greatly diminish technical barriers and complications that could interfere with the success of the program.

The Rare Bone Disease TeleECHO offers 1 h of American Medical Association Physician Recognition Award Category 1 Continuing Medical Education credit per session at no cost to attendees through the University of New Mexico School of Medicine Office of Continuing Medical Education and Professional Development. Completion of a short post-session evaluation is required to obtain CME credit.

## Experience to Date

**Fig. 2:**
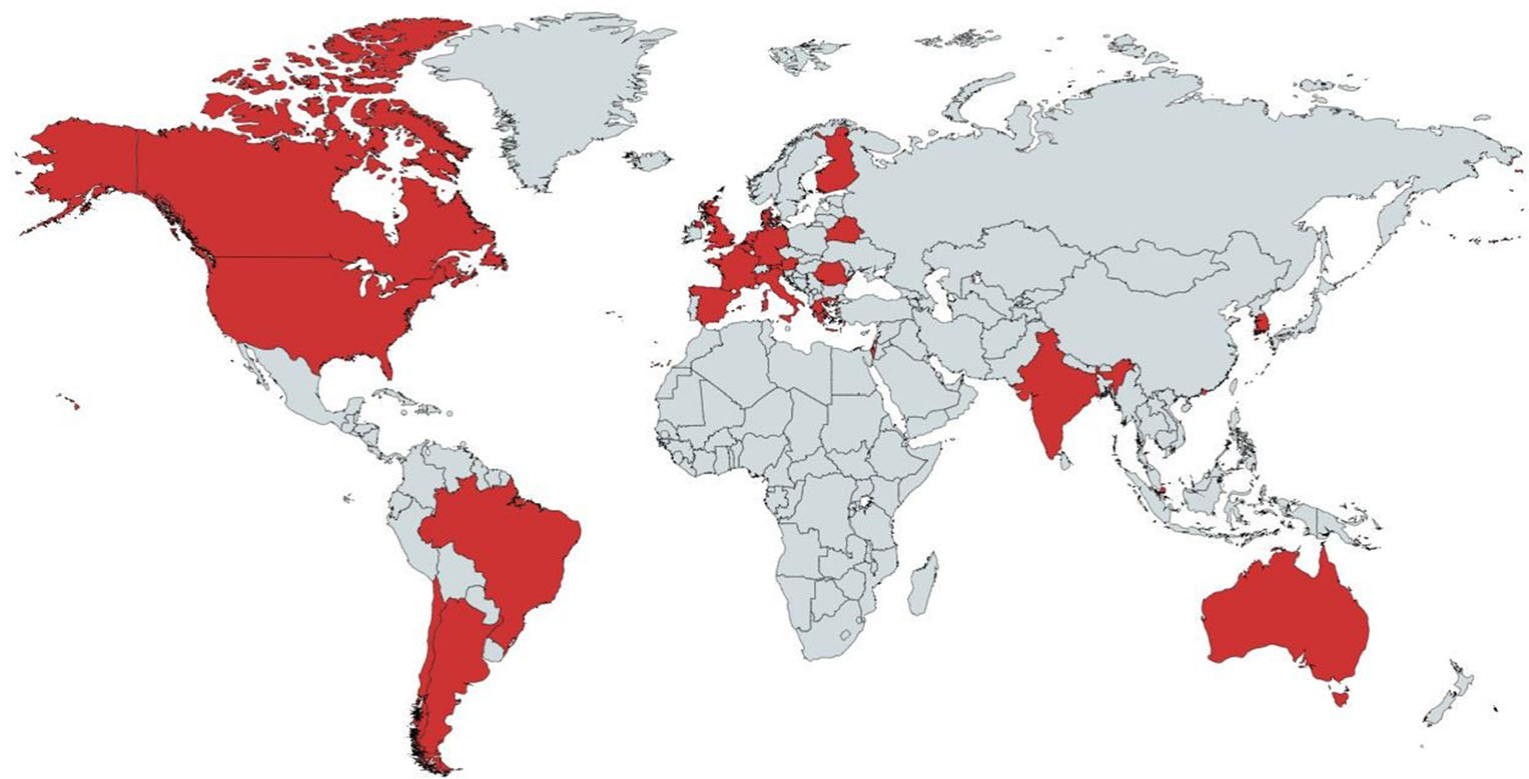
Global reach. The Rare Bone Disease TeleECHO has had an impact on clinicians outside the USA. To date, 24 countries (including the United States) marked in red have participated in TeleECHO sessions

Evaluation surveys and attendance records from the first 8 months of hosting the Rare Bone Disease TeleECHO reveal that the program has drawn 330 unique registrants from 24 different countries. Seventy-five registrants (23%) have participated from outside the USA. The first nine programs have already garnered a total of 1440 views on YouTube. Our global reach is one of the highlights of our program and supports the Project ECHO goal of “democratizing” healthcare (Fig. 2).

Although all Rare Bone Disease TeleECHO sessions focus on disorders with significant skeletal manifestations, there is wide variation in the prevalence and clinical presentations of rare bone disorders. For this reason, the attendees who chose to participate change every month. Qualitative data elicited from evaluation surveys identify three themes. First, all attendees report an increased awareness of the symptoms, clinical pearls, diagnostic criteria, and management of rare bone diseases. Second, attendees indicate an increased level of confidence as healthcare providers due to their expanded skillset. Third, some attendees note that the ECHO session was their first-ever encounter with some of the presentation topics.

As we approach the end of the inaugural year of the Rare Bone Disease TeleECHO, a few lessons have been learned that are critical for other groups who are thinking about starting their own program. First, it is essential to have a dedicated support staff member. The many tasks involved in hosting a TeleECHO session such as documenting conflicts of interest, ensuring that presentations are appropriate for CME credit, advertising sessions, soliciting case presentations, moderating Zoom teleconferences, collecting data from evaluation surveys, and administering CME credit necessitates 0.25–0.5 FTE financial support. In addition, although our program was intended for orthopaedic surgeons, endocrinologists, nephrologists, and geneticists, we have seen a broader array of participants than originally anticipated: pharmaceutical representatives, FDA employees, physical therapists, and midlevel practitioners. We believe that this underscores the need for rare bone disease education outside of traditional venues and supports our efforts to emphasize differential diagnoses and utilization of technology.

Registration for the Rare Bone Disease TeleECHO is available at https://oif.org/research/echo. Recordings of each session can be viewed on the same OIF website. Our faculty are currently preparing a curriculum for the second year of the Rare Bone Disease TeleECHO, which is scheduled to begin in August 2020 **(**Table 2**).** The proof-of-concept experience and success of the Rare Bone Disease TeleECHO has inspired an offshoot program focused on osteogenesis imperfecta which is slated to launch in the fall of 2020.

**Table 2:** Rare Bone Disease TeleECHO Agenda Year Two

8/6/2020	Melorheostosis: The Genes Behind the Dripping Candle Wax	Timothy Bhattacharyya, MD – National Institutes of Health, NIAMS
9/3/2020	Evaluation of Patients with Hyperphosphatemia	Michael Collins, MD – National Institutes of Health, NIDCR
10/1/2020	Mechanisms of Bone Loss in Complex Lymphatic Anomalies	Michael Kelly, MD, PhD – Northeast Ohio Medical University
11/5/2020	Dental Concerns in Patients with Rare Bone Disorders	Tim Wright, DDS, MS – University of North Carolina
12/3/2020	Generalized Arterial Calcification of Infancy (GACI)	Carlos Ferreira, MD – National Institutes of Health, NHGRI
1/7/2021	Skeletal Surveys – A Systematic Approach	Dorothy Bulas, MD – Children’s National Hospital
2/4/2021	Jansen’s Disease	Harald Jueppner, MD – Massachusetts General Hospital
3/4/2021	Bone Pain in Children	Alison Boyce, MD – National Institutes of Health—NIDCR
4/1/2021	Multiple Hereditary Exostoses	David S. Feldman, MD – St. Mary’s Medical Center
5/6/2021	DXA Evaluation in the Child	Catherine Gordon, MD – Boston Children’s Hospital
6/3/2021	Adult Hypophosphatasia	Kathryn Dahir, MD – Vanderbilt University Medical Center
7/1/2021	Evaluation of the Child with Multiple Fractures	Eric T. Rush, MD – University of Kansas Medical Center

## Summary

It is estimated that the entire body of medical knowledge will double every 73 days in 2020 compared with every 7 years in 1980 and every 50 years in 1950 [4]. What medical students learned in their first 3 years of training a decade ago only comprises 6% of what is known today [4]. Further, medical knowledge tends to be centralized at major academic institutions and large cities, while smaller clinics are forced to rely on limited real-world evidence and sometimes outdated treatment modalities. These clinics also do not always have access to the multidisciplinary team support needed to manage complex conditions that large institutions have at their disposal.

Innovations in diagnosis and management of all rare disorders are proliferating rapidly; the challenge for specialty—and community—physicians is keeping up with these developments. The Rare Bone Disease TeleECHO program helps meet that challenge by offering a strong connection to the research enterprise for all interested practitioners with a learning environment centred on actual cases. Participants can present their cases to, and receive feedback from, leading practitioners, researchers, and their own peers. This exchange of expert knowledge democratizes and multiplies the impact that the research enterprise can have on patient outcomes. Moreover, the Rare Bone Disease TeleECHO supports optimal retention of information through case-based, interactive learning instead of a lecture format [5, 6]. Evaluation surveys confirm that the Rare Bone Disease TeleECHO program is meeting its goals and objectives to enhance the skill sets of clinicians treating rare bone diseases. The experience of the Rare Bone Disease TeleECHO provides insights for other rare disease groups on how they, too, can extrapolate the ECHO model to meet their needs.

## Data Availability

Not applicable.
